# Advancing Near-Infrared Light Sources: Enhancing Chromium Emission through Cation Substitution in Ultra-Broadband Near-Infrared Phosphors

**DOI:** 10.1021/acs.chemmater.3c02466

**Published:** 2023-11-17

**Authors:** Natalia Majewska, Yi-Ting Tsai, Xiang-Yun Zeng, Mu-Huai Fang, Sebastian Mahlik

**Affiliations:** † Institute of Experimental Physics, Faculty of Mathematics, Physics and Informatics, University of Gdansk, Wita Stwosza 57, 80-308 Gdansk, Poland; ‡ Research Center for Applied Sciences, Academia Sinica, Taipei 11529, Taiwan; § International Centre for Theory of Quantum Technologies (ICTQT), University of Gdansk, 80-308 Gdansk, Poland

## Abstract

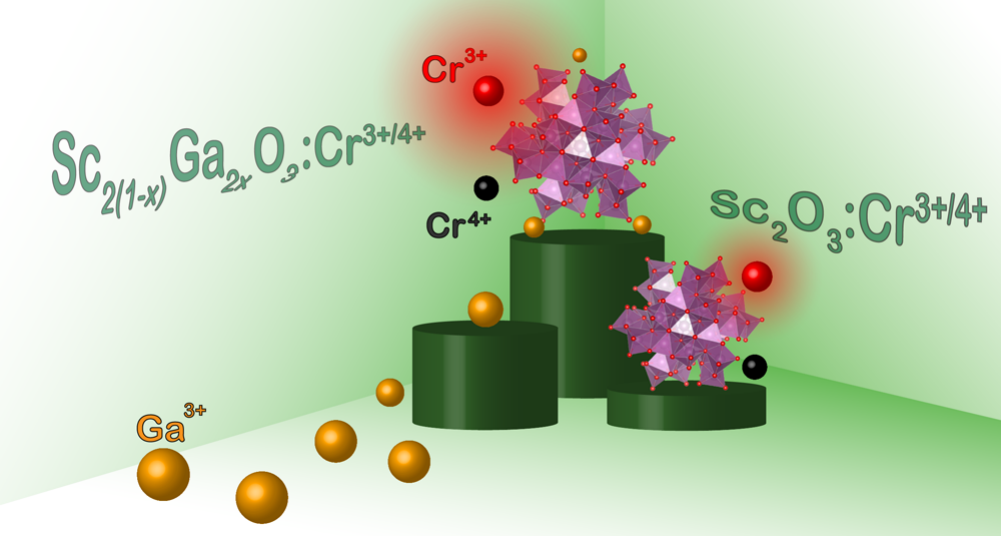

The growing interest in the use of near-infrared (NIR) radiation for spectroscopy, optical communication, and medical applications spanning both NIR-I (700–900 nm) and NIR-II (900–1700 nm) has driven the need for new NIR light sources. NIR phosphor-converted light-emitting diodes (pc-LEDs) are expected to replace traditional lamps mainly due to their high efficiency and compact design. Broadband NIR phosphors activated by Cr^3+^ and Cr^4+^ have attracted significant research interest, offering emission across a wide range from 700 to 1700 nm. In this work, we synthesized a series of Sc_2(1–*x*)_Ga_2*x*_O_3_:Cr^3+/4+^ materials (*x* = 0–0.2) with broadband NIR-I (Cr^3+^) and NIR-II (Cr^4+^) emission. We observed a substantial increase in the intensity of Cr^3+^ (approximately 77 times) by incorporating Ga^3+^ ions. Additionally, our investigation revealed that energy transfer occurred between Cr^3+^ and Cr^4+^ ions. Configuration diagrams are presented to elucidate the behavior of Cr^3+^ and Cr^4+^ ions within the Sc_2_O_3_ matrix. We also observed a phase transition at a pressure of 20.2 GPa, resulting in a new unknown phase where Cr^3+^ luminescence exhibited a high-symmetry environment. Notably, this study presents the pressure-induced shift of NIR Cr^4+^ luminescence in Sc_2(1–*x*)_Ga_2*x*_O_3_:Cr^3+/4+^. The linear shifts were estimated at 83 ± 3 and 61 ± 6 cm^–1^/GPa before and after the phase transition. Overall, our findings shed light on the synthesis, luminescent properties, temperature, and high-pressure behavior within the Sc_2(1–*x*)_Ga_2*x*_O_3_:Cr^3+/4+^ materials. This research contributes to the understanding and potential applications of these materials in the development of efficient NIR light sources and other optical devices.

## Introduction

The growing interest in the use of near-infrared (NIR) radiation, spanning both NIR-I (700–900 nm) and NIR-II (900–1700 nm), stems from its vast and remarkable applications in fields such as food science, security, and biomedicine. Consequently, there is an urgent need to address the development of a new generation of NIR light sources.^1−4^ As a new generation of NIR light sources, NIR phosphor-converted light-emitting diodes (pc-LEDs) are anticipated to replace traditional tungsten halogen lamps due to their low cost, high efficiency, and compact design.^5^ These pc-LEDs utilize commercial high-power blue LED chips in combination with inorganic phosphors to achieve the desired near-infrared wavelength. Phosphors activated by transition metals (such as Cr^3+^, Fe^3+^, Mn^2+^, and Ni^2+^)^6−13^ and lanthanide ions (including Yb^3+^, Er^3+^, and Eu^2+^)^14−20^ are considered up-and-coming candidates for the new generation of NIR pc-mini/micro-LEDs. Additionally, Bi^3+^-activated materials^21^ and zero-dimensional hybrid antimony chlorides^22^ stand out as intriguing alternatives for NIR applications. These NIR light sources can be easily integrated into smartphones or wearable devices, enabling a wide range of functional applications.

In recent years, Cr^3+^-activated broadband NIR phosphors have garnered significant research interest owing to their exceptional characteristics, including high efficiency, high thermal stability, and tunable spectra.^23−28^ These phosphors offer a broad range of emission wavelengths in the near-infrared region. Additionally, Cr^4+^ has emerged as a promising luminescent center with broadband emission spanning from 1100 to 1700 nm. There are indeed numerous examples of broadband NIR Cr^4+^-activated phosphors, some of which include Li_2_ZnGeO_4_:Cr^4+^,^29^ Li_2_CaGeO_4_;Cr^4+^,^30^ Y_3_Al_5_O_12_:Cr^4+^,^31^ and Y_2_SiO_5_:Cr^4+^,^32,33^ as well as the coexistence of Cr^3+^ and Cr^4+^ in MgSiO_4_,^34,35^ and CaGa_4_O_._^36,37^ Recently, Wang et al.^18^ demonstrated the potential application of Sc_2_O_3_:Cr^3+^,Cr^4+^ phosphor as temperature sensors. By codoping Yb^3+^ ions, they achieved a continuous NIR ultra-broadband emission spectrum from 650 to 1600 nm. This development opens up possibilities for utilizing these phosphors in spectral analysis applications.

Scandium oxide (Sc_2_O_3_) holds promising potential as a host material for the incorporation of luminescent dopants. Sc_2_O_3_ is regarded as a rare-earth sesquioxide because of similar chemical behavior.^38^ Until now, researchers have identified five distinct structural modifications in rare-earth sesquioxides.^39−41^ Three phases are designated as follows: hexagonal phase with space group *P*3*m*1 and seven-coordinated cations (A phase); monoclinic phase with space group *C*2*/m*, where each cation is surrounded by six or seven anions (B phase); and cubic phase with space group *Ia*3̅ with six-coordinated cations (C phase). These phases are observed at room temperatures and atmospheric pressures. The two other phases are formed at very high temperatures: H phase (hexagonal, *P*6_3_*/mmc*) and X phase (cubic, Im3̅*m*).^42^ Yusa et al. noted that under high pressures and temperatures the B phase of Sc_2_O_3_ undergoes the phase transition to the Gd_2_S_3_ structure.^43^ The irreversible phase transition from cubic space group *Ia*3̅ (referred to as the *C* phase*)* to monoclinic space group *C*2/*m* (*B* phase) is calculated to occur at 15 GPa. The same research group has experimentally shown it to occur at 32 GPa.^44^ Other experimental studies show that phase transitions occur at 25–28 GPa.^45^ Yusa et al.^43^ found that the corundum phase, not previously seen in Sc_2_O_3_, can only be synthesized as a recovered product from the Gd_2_S_3_ phase.

Introducing Cr^3+^ ions into the Sc_2_O_3_ matrix leads to the generation of a broad emission band within the NIR-I region. Furthermore, the presence of Cr^4+^ ions in the matrix also gives rise to emission within the NIR-II region. Several studies have investigated the luminescence properties of pure Sc_2_O_3_:Cr^3+^.^46−48^ However, there is a lack of research specifically focusing on mixed-ion materials. The modification of the matrix through cation substitution enables the tuning of emission characteristics to meet the specific application requirements.

In the previous study, we investigated the new Ga_2(1-y)_Sc_2*y*_O_3_ materials synthesized in the monoclinic crystal structure with the space group *C*2*/m*.^49^ We showed that *y* could reach around 0.44 and Sc could not be doped more in the structure. In this study, we demonstrate that even though Sc cannot be doped in the Ga_2_O_3_ structure for more than 44%, it is still possible to incorporate Ga into the Sc_2_O_3_ structure.

Understanding the luminescence mechanisms of Sc_2_O_3_ codoped with Cr^3+^ is essential for further optimizing its luminescence properties for commercial applications. This study focuses on investigating the luminescence properties of the Sc_2(1–*x*)_Ga_2*x*_O_3_:Cr^3+/4+^ solid solution. We will examine the thermal analysis of this material and explore the influence of the crystal field effects on the optical properties of Cr^3+^ and Cr^4+^ ions.

## Results and Discussion

### Structural Analyses

**Figure 1:**
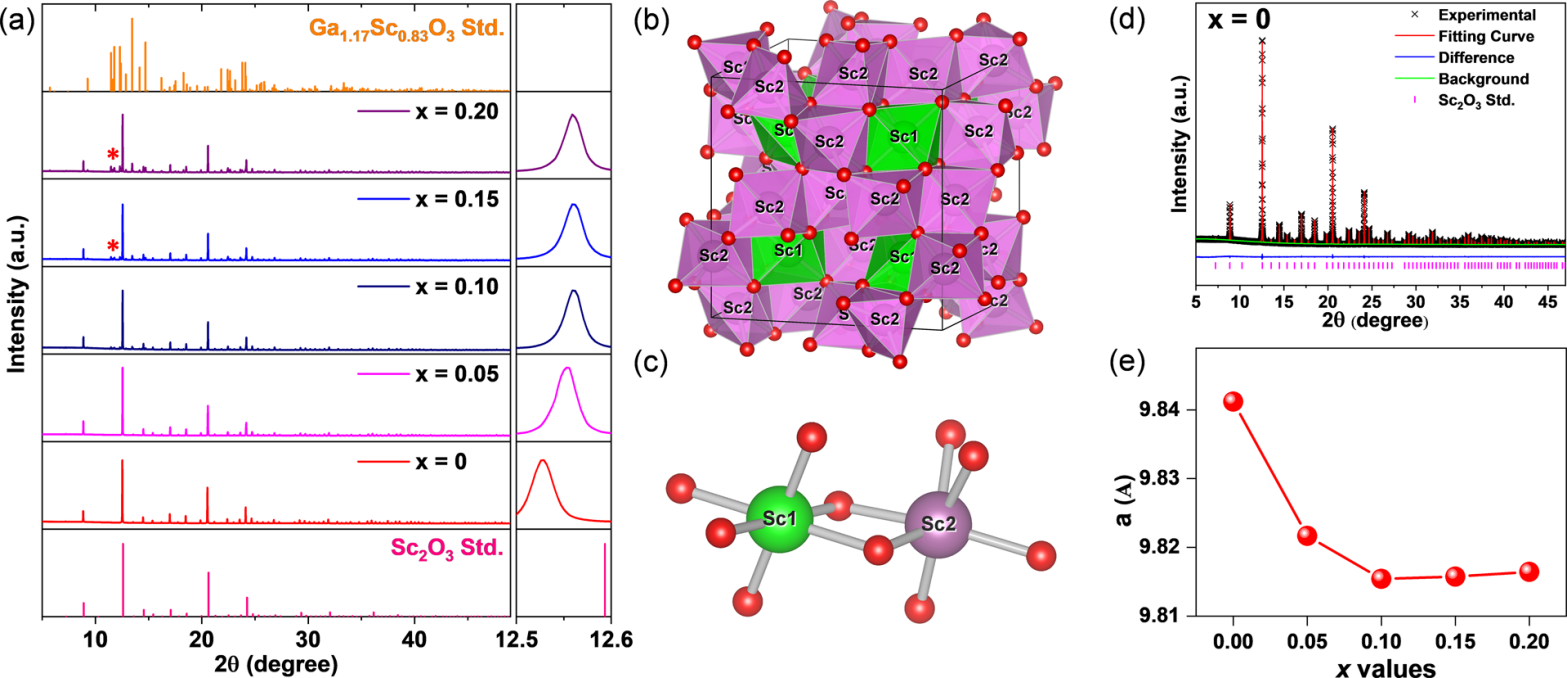
(a) XRD patterns of the SGOC with *x* = 0–0.2. (b) Crystal structure of Sc_2_O_3_. (c) Coordinated environment of Sc1 and Sc2 sites in Sc_2_O_3_. (d) Rietveld refinement of the SGOC with *x* = 0. (e) Lattice parameters of SGOC with *x* = 0–0.2. The asterisk symbol in (a) indicates the diffraction peaks from the Ga_1.17_Sc_0.83_O_3_ impurity phase.

The synchrotron X-ray powder diffraction of Sc_2(1–*x*)_Ga_2*x*_O_3_:Cr^3+/4+^ (SGOC) with *x* = 0–0.20 is characterized, as shown in Figure 1a. No impurity peaks are found for *x* = 0–0.05, and only slight impurity peaks are detected for *x* = 0.10. By contrast, more impurities could be seen for *x* = 0.15–0.20, as shown by the asterisk symbol in Figure 1a. The impurity can be attributed to Ga_1.17_Sc_0.83_O_3_ because the position of the impurity peak aligns with the standard peak position of the Ga_1.17_Sc_0.83_O_3_ phase attracted from ICSD-422271. For the *x* = 0–0.10 samples, the diffraction peaks shift toward higher angles because the ionic radius of Sc^3+^ (0.745 Å; CN = 6) (CN denotes coordinated number) is bigger than that of Ga^3+^ (0.62 Å; CN = 6).^50^ Notably, the diffraction peak position only shifts to higher angles for *x* = 0–0.10 samples, and it does not shift for *x* = 0.10–0.20, indicating that Ga^3+^ ions cannot be incorporated into the Sc_2_O_3_ structure anymore. The structural information and standard XRD diffraction patterns of Sc_2_O_3_ and Ga_1.17_Sc_0.83_O_3_ are extracted from the crystallographic information framework (CIF) with COD-1008928 and ICSD-422271, respectively. Ga_1.17_Sc_0.83_O_3_ possesses the same structure as Ga_2_O_3_, as shown in Figure S1 in the Supporting Information (SI).

The crystal structure of Sc_2_O_3_ is shown in Figure 1b. Sc_2_O_3_ crystallizes in a cubic structure with the space group of *Ia*3̅ (no. 206). There are two Sc^3+^ sites in the structure, and both are coordinated by six O^2–^ ions to form 6-fold polyhedrons, as shown in Figure 1c. The detailed coordination environment of Sc1 and Sc2 in [ScO_6_] polyhedrons is shown in Table S1 in SI. The bond lengths of the six Sc1–O1 bonds are identical and equal to 2.10314 Å. On the other hand, the six Sc2–O1 bonds are different, with an average bond length of 2.0978 Å. The distortion index can be utilized to realize the bond length deviation by the following formula^51^:
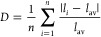
1where *D* is the distortion index; *l*_av_ is the average bond length; *l*_*i*_ is the bond length between the center ion and *i*th coordinated ions; *n* is the number of the coordinated ions. The calculated *D* values of Sc1 and Sc2 sites equal 0 and 0.00604, respectively. Despite the small *D* of the Sc2 site, the coordinated environment between the Sc1 and Sc2 sites is different. As shown in Table S1 in the SI, four O^2–^ ions are in the same plane, while the other two O^2–^ ions are offset from the vertical direction for the Sc1 site. On the contrary, despite the 6-fold coordination, the coordinated environment of the Sc2 site differs from the regular octahedron coordination, which is more distorted. One cannot observe four O^2–^ ions in the same plane around the Sc2 site. To quantify the degree of distortion in bond angles, the bond angle variance can be calculated by the following equation^52^:
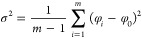
2where σ^2^ is the bond angle variance; *m* is the number of bond angles, which equals the number of faces ×3/2; φ_*i*_ is the *i*th bond angle; and φ_0_ is the ideal bond angle for the selected polyhedron, which equals 90° for an octahedron. σ^2^ calculated for Sc1 and Sc2 are 180.5362 and 246.4595 (degree),^2^ respectively, indicating that the coordinated environment of Sc2 is much more distorted than that of Sc1. Although the bond angle variance in the Sc1 site is less than the Sc2 site, it is still much more than most of the octahedral coordination. Take the Ga_2_O_3_ and Ga_1.17_Sc_0.83_O_3_ structures as examples, the bond angle variances calculated from their CIF are 41.9175 and 48.2954, respectively. This result indicates that the [CrO_6_] polyhedrons in Sc_2_O_3_ will still be more distorted than [CrO_6_] polyhedrons in Ga_2_O_3_ and Ga_1.17_Sc_0.83_O_3_. The bond angle variance is beneficial in analyzing the distortion behavior and compensates for the disadvantage of the distortion index, which considers the bond length. One should note that the electronic transition for the Cr ion belongs to *d*–*d* transitions, for which the bond angles could potentially affect the luminescent properties.

To further understand the structural properties, the Rietveld refinements of SGOC with *x* = 0–0.20 are conducted, as shown in Figures 1d and S2 in the SI. The refined parameters, atomic positions, occupancies, and displacement parameters of SGOC with *x* = 0–0.20 are shown in Tables S2 and S3. The experimental data fit well with the Sc_2(1–*x*)_Ga_2*x*_O_3_ patterns. As expected, the lattice parameter, *a*, decreases in *x* = 0–0.10 and stays nearly constant in *x* = 0.10–0.20, as shown in Figure 1e. In our previous study, we have achieved the pure phase of Ga_2(1–*y*)_Sc_2*y*_O_3_:Cr^3+^ from the Ga_2_O_3_ structure as the Sc^3+^/Ga^3+^ ratio is lower than 44%, as shown in Figure S3 in SI.^49^ By contrast, in this study, we can obtain the pure phase of Sc_2(1–*x*)_Ga_2*x*_O_3_ from the Sc_2_O_3_ structure as the Sc^3+^/Ga^3+^ ratio is higher than 94%. Furthermore, there is no significant preference for Ga^3+^ ions when incorporated into the Sc1 and Sc2 sites, as shown in Table S3.

### Photoluminescence Analysis

**Figure 2:**
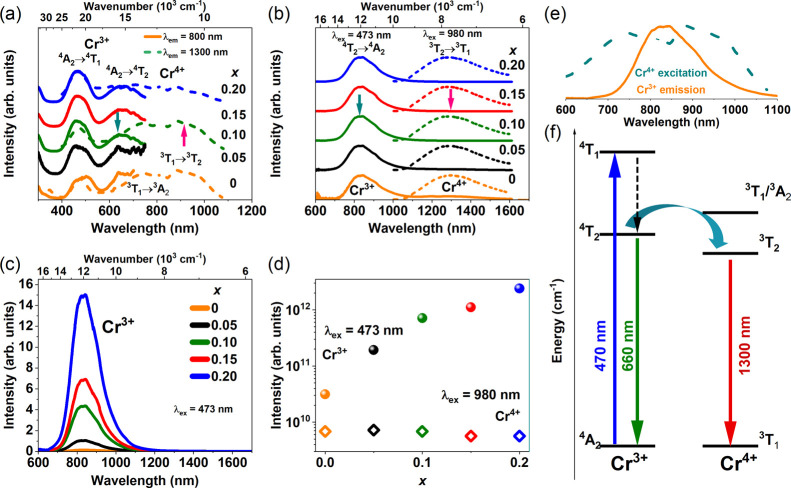
Room temperature (a) PLE spectra upon Cr^3+^ and Cr^4+^ observation at 800 and 1300 nm, respectively, and (b) normalized PL spectra upon excitation at 473 (Cr^3+^) and 980 nm (direct Cr^4+^ excitation). (c) Unnormalized room temperature PL spectra upon excitation at 473 nm. (d) Arbitral emission intensity of Cr^3+^ (dots) and Cr^4+^ (diamonds) emission. (e,f) Evidence of the energy transfer mechanism.

To examine the basic optical properties, room temperature (RT) photoluminescence excitation (PLE) and photoluminescence (PL) spectra of SGOC for *x* = 0–0.20 are shown in Figure 2a,b, respectively. Upon NIR emission observation at 800 nm, the PLE spectra consist of two excitation bands typical for Cr^3+^ ions in 6-fold octahedral coordination. The high energy band that peaked at 470 nm corresponds to ^4^A_2_ → ^4^T_1_ transition, while the lower energy band that peaked at 660 nm is related to the ^4^A_2_ → ^4^T_2_ transitions of Cr^3+^ ions. Upon excitation at 473 nm, a broadband emission extending from 650 to 1100 nm (NIR-I) with a maximum at 830 nm is observed (Figure 2b). This emission corresponds to the ^4^T_2_ → ^4^A_2_ spin-allowed transition of Cr^3+^ ions in a weak crystal field. Excitation at 450 nm effectively stimulates NIR-I Cr^3+^ emission, suggesting its potential application in NIR-LEDs. Additionally, the less intense band appears at a longer wavelength (NIR-II), 1100–1600 nm, as shown in Figure S4a in SI. This emission can also be excited by 980 nm, and the corresponding emission spectra are shown in Figure 2b with a dashed line. Kück et al.^47^ observed identical emission spectra for Sc_2_O_3_:Cr^3+^. The RT full width at half-maximum (*fwhm*) values for Cr^3+^ and Cr^4+^ ions are 2402 and 2075 cm^–1^, respectively. Figure 2a shows the excitation spectra of the NIR-II emission observation at 1300 nm (represented by the dashed line). The two excitation bands at 470 and 660 nm correspond to the excitation of Cr^3+^ ions, indicating energy transfer between NIR-I (Cr^3+^) and NIR-II (Cr^4+^). Two additional overlapped bands, extending from 650 to 1050 nm, are observed in the NIR-II excitation spectra. Although most papers show that Cr^4+^ ions prefer to occupy tetrahedral sites,^30,32,34,37,53^ some studies^54^ show that Cr^4+^ can also occupy the octahedrally coordinated sites. Both 6-fold coordinated Sc1 and Sc2 sites are distorted, but the distortion of Sc2 sites is much more significant. We can consider the 6-fold coordinated Sc1 site as a distorted octahedron. In contrast, the Sc2 site cannot be regarded as a typical octahedron, and the Cr^4+^ ions may occupy this strongly distorted 6-fold coordinated Sc2 site. The most likely NIR-II emission is related to the Cr^4+^ ions in disordered 6-fold coordination. According to the Tanabe-Sugano diagram for *d*^2^ electron configuration,^55,56^ 650–1050 nm excitation bands can be attributed to the ^3^T_1_ → ^3^T_2_ and ^3^T_1_ → ^3^A_2_ (or ^3^T_1_ → ^3^T_1_) transitions of Cr^4+^ ions. Hence, the emission extending from 1100–1600 nm with a maximum of 1300 nm corresponds to the ^3^T_2_ → ^3^T_1_ emission of Cr^4+^.

Figure S4b shows the *x*-dependent position of the ^4^A_2_ → ^4^T_1_,^4^T_2_, and ^4^T_2_ → ^4^A_2_ transitions of Cr^3+^ and ^3^T_2_ → ^3^T_1_ of Cr^4+^ in SGOC (Sc_2(1–*x*)_Ga_2*x*_O_3_:Cr^3+/4+^). A minimal blue shift of the broadband emission of Cr^3+^ and Cr^4+^ ions and excitation bands of Cr^3+^ ions is observed with an increase in the *x* value of up to 0.10. The emission band shift should be interpreted based on the crystal field theory, assuming that incorporating smaller Ga^3+^ in place of Sc^3+^ ions results in an increase in the crystal field strength *Dq* in the vicinity of Cr^3+^/Cr^4+^ ions. This leads to an increase in the energy of the ^4^T_1_ and ^4^T_2_ states with respect to the ^4^A_2_ ground state for Cr^3+^ ions. Similarly, for Cr^4+^ ions, the energy of ^3^T_2_ and ^3^A_2_ (or ^3^T_1_) increases with respect to the ^3^T_1_ ground state. In other words, this shift is due to the increase in the crystal field with increasing Ga concentration, which is also confirmed by XRD. The XRD measurements clearly demonstrate that Ga^3+^ ions are incorporated into the crystal structure only up to *x* = 0.10. Consequently, the increase in crystal field strength is limited to *x* = 0.10, and there is no further change for higher *x* values. As a result, the shift in emission and excitation bands is solely observable up to *x* = 0.10. The crystal field parameter *Dq* and Racah parameters *B* and *C* for Cr^3+^ are discussed in SI.

Figure 2c shows arbitral emission intensity upon excitation at 473 nm, while Figure 2d shows the integrated emission intensities of Cr^3+^ and Cr^4+^ for different *x* concentrations upon excitation at 473 and 980 nm, respectively. The intensity of Cr^3+^ emission increases significantly (around 77 times) from *x* = 0 to *x* = 0.20. In the case of Cr^4+^ emission, the intensity hardly changes and only slightly decreases for samples with a higher *x* concentration. Additionally, the photoluminescence spectra at 100 K in Figure S4c in SI show that the relative intensity of Cr^3+^ to Cr^4+^ increases with *x*. The ionic radius of Sc^3+^ (0.745 Å) is indeed bigger than that of Cr^3+^ (0.615 Å). Additionally, the ionic radius of Cr^3+^ is similar to that of Ga^3+^ (0.62 Å). These variations in ionic radii lead to a decrease in the average size of the octahedra, potentially facilitating the doping of Cr^3+^ ions. As a result, in a Ga^3+^-doped sample, Cr^3+^ may exhibit a higher likelihood of occupying these sites, which can contribute to an increase in the intensity of luminescence.

As shown in Figure S4d, the decay profiles of Cr^3+^ luminescence are multiexponential for materials without Ga (*x* = 0) and for low *x* concentration, and they start to become near single-exponential for materials with higher *x* (Ga) concentration. The opposite behavior could be expected: increasing multiexponential behavior with increasing *x* due to the different surroundings around the Cr^3+^ ion. The observed effect on the luminescence kinetics suggests a decrease in energy transfer with increasing *x*. The increase in the intensity of Cr^3+^ luminescence in a Ga^3+^-doped sample can indeed lead to a situation where the observability of energy transfer decreases in the decay profile. This may suggest that the energy transfer remains consistent across all samples, as evidenced by only slight shifts in the excitation and emission spectra, which should not significantly change the energy transfer efficiency. The heightened prominence of Cr^3+^ emissions can give the impression of a more single-exponential decay even when multiple energy transfer pathways might be involved. This effect arises because the contribution of the energy transfer process to the decay profile becomes relatively weaker in the presence of stronger Cr^3+^ luminescence.

Because of the multiexponential behavior, the average decay time was calculated for all studied materials using the equation:
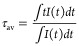
3where *I*(*t*) is the luminescence intensity at time *t*. The calculated average decay times of Cr^3+^ ions are shown in Figure S4e. The average decay time increases with increasing *x*, ranging from approximately 17 to 37 μs. The decay profiles of Cr^4+^ shown in Figure S4f in the SI are relatively similar for all samples. The calculated average decay time using eq 3 is approximately 0.42 μs, 2 orders of magnitude shorter than the decay time of Cr^3+^ ions.

For a closer look at the energy transfer mechanism, the Cr^4+^ PLE spectra and the Cr^3+^ PL spectra are presented in Figure 2e. It can be observed that the Cr^3+4^T_2_ → ^4^A_2_ emission overlaps with the Cr^4+^ excitation bands. This overlapping implies the potential for energy transfer from the lowest excited ^4^T_2_ state of the Cr^3+^ ion to the ^3^T_2_ or ^3^T_2_/^3^A_2_ excited states of the Cr^4+^ ions. Consequently, the NIR-II luminescence from ^3^T_2_ to ^3^T_1_ of Cr^4+^ is observed as shown in Figure 2f.

### Oxidation State of the Chromium Ions

**Figure 3:**
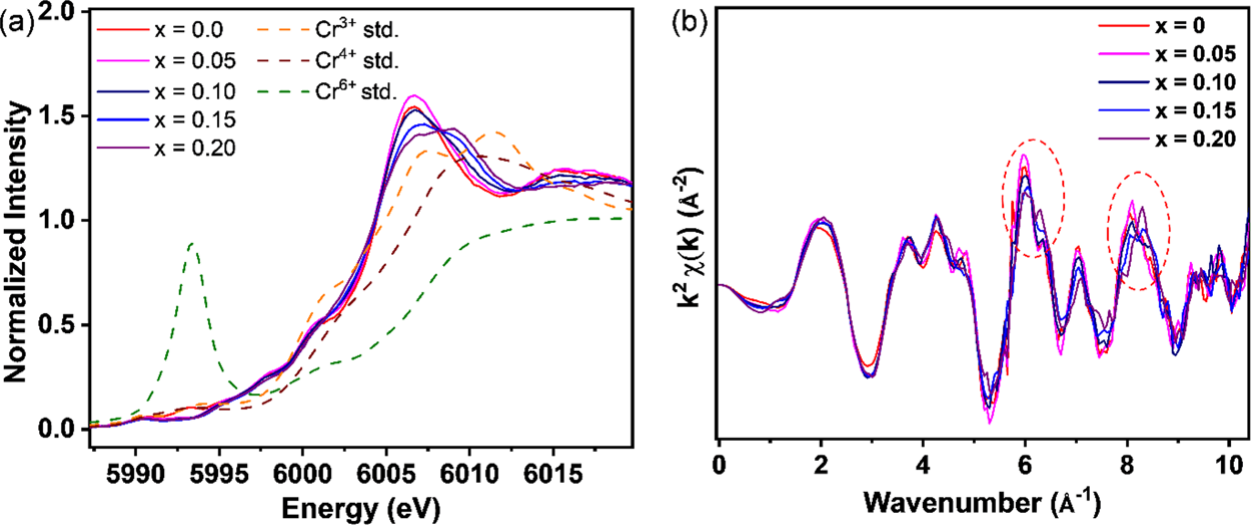
Cr *K*-edge XANES spectra and (b) Cr *K*-edge *k*^2^-weighted Fourier transforms of EXAFS spectra.

The oxidation state of the activators will significantly affect the luminescent properties. To determine the oxidation state of the activators in the bulk powder materials, the Cr *K*-edge X-ray absorption near edge structure (XANES) spectra of the SGOC with *x* = 0–0.20 are measured, as shown in Figure 3a. All the absorption edges of *x* = 0–0.20 samples are close to the standard patterns of Cr^3+^ and Cr^4+^, while they differ from the standard patterns of Cr^6+^. This effect reveals the possibility of Cr^3+^ and Cr^4+^ coexisting in the SGOC phosphors. Besides, there is no intense pre-edge peak at around 5,993 eV, proving the lack of Cr^6+^ in the materials. On the other hand, the peak shape of *x* = 0–0.10 samples of peak maximum at around 6,007 eV is similar, while the ones of *x* = 0.15–0.20 samples are different. This result indicates that Cr may exist in Ga_1.17_Sc_0.83_O_3_ and contribute to the XANES spectra when *x* is higher than 0.15. Furthermore, the Cr *K*-edge *k*^2^-weighted Fourier transform of extended X-ray absorption fine structure (EXAFS) spectra of the SGOC with *x* = 0–0.20 is analyzed, as shown in Figure 3b. The oscillation patterns between them are roughly similar; however, one can still observe the subtle change in oscillation patterns for *x* = 0.15–0.20 samples, supporting the hypothesis that the Cr in Ga_1.17_Sc_0.83_O_3_ may contribute to XANES and EXAFS.

### Temperature-Dependent Photoluminescence

Measurements were conducted while the temperature was varied to enhance comprehension of both radiative and nonradiative processes. Due to the presence of impurities in *x* = 0.15–0.20 samples, the subsequent analysis of luminescent property in this study will mainly focus on the *x* = 0 and 0.10 samples.

**Figure 4:**
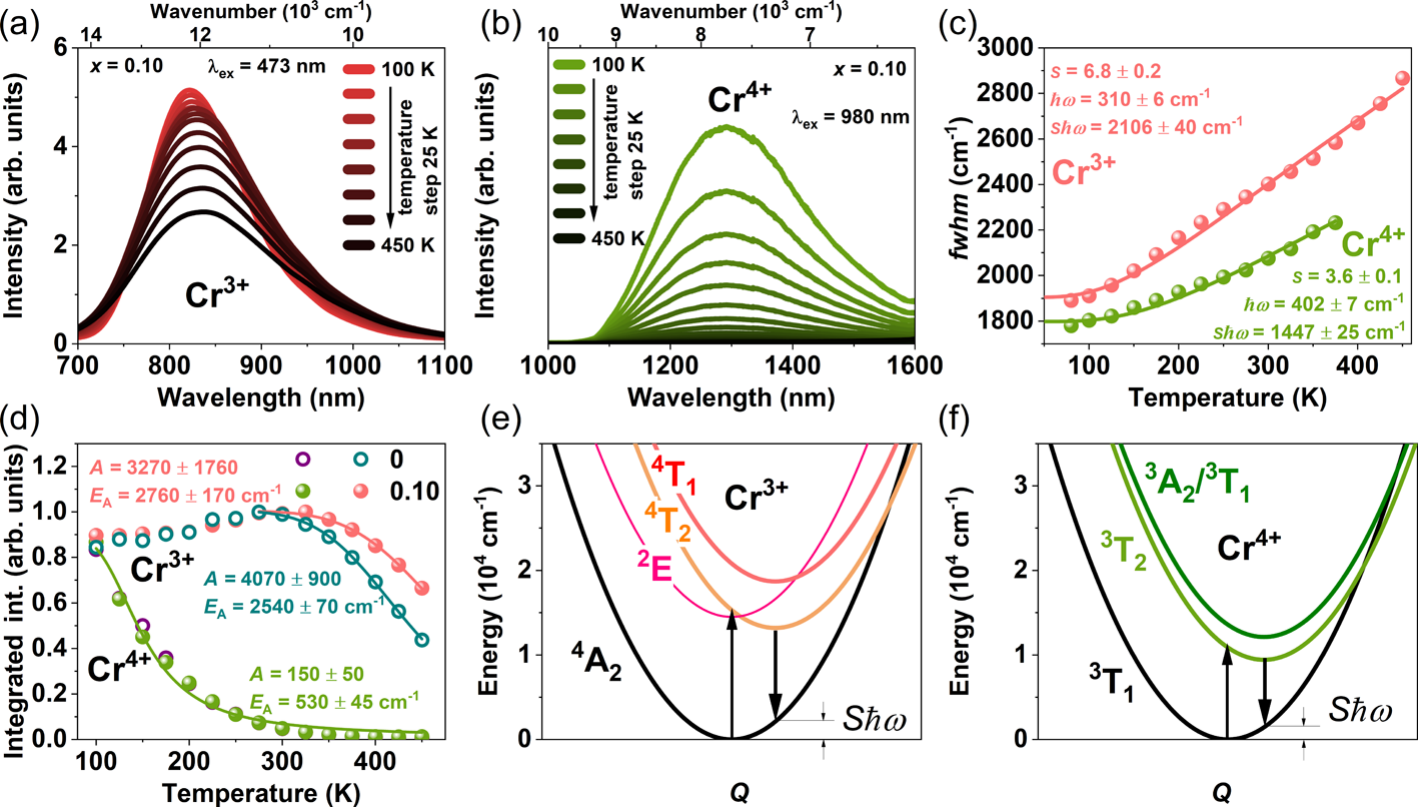
Temperature dependence of the luminescence of Cr^3+^ and Cr^4+^ in SGOC. The temperature-dependent emission spectra are (a) upon excitation at 473 nm for Cr^3+^ and (b) at 980 nm for Cr^4+^ for *x* = 0.10. (c) Temperature dependence of *fwhm* for *x* = 0 and 0.10. (d) Total emission intensity of Cr^3+^ and Cr^4+^ for *x* = 0 and 0.10. The purple circles and green dots represent Cr^4+^ in *x* = 0 and *x* = 0.10, respectively. The cyan circles and red dots represent Cr^3+^ in *x* = 0 and *x* = 0.10, respectively. Solid lines represent the fitting to the experimental data using formulas (4 and 5). Configuration coordinate diagrams for the (e) Cr^3+^ and (f) Cr^4+^ ions for *x* = 0.10.

Figure 4a,b shows the temperature-dependent steady-state PL emission of Cr^3+^ ions upon excitation at 473 nm and Cr^4+^ ions upon excitation at 980 nm for *x* = 0.10, respectively. To mitigate the overlap of Cr^3+^ emission with Cr^4+^ emission, we decided to investigate the temperature dependence of the emission intensity of Cr^4+^ under near-infrared 980 nm excitation. The measurements were performed in the temperature range of 100–450 K. The temperature-dependent luminescence of Cr^3+^ and Cr^4+^ for *x* = 0 is shown in Figure S5a,b, respectively. For both samples, emission bands become broadened with increasing temperature. The temperature dependence *fwhm* of the Cr^3+^ (represented by red dots) and Cr^4+^ (represented by green dots) emissions for *x* = 0.10 is shown in Figure 4c. For temperatures exceeding 400 K, the emission intensity becomes too weak to accurately determine the *fwhm* of the Cr^4+^ emission. The *fwhm* value increases with an increasing temperature. We have utilized the hyperbolic cotangent law describing the temperature dependence of the emission band (*fwhm)* given by equation:^57^
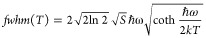
4where *T* is temperature, *S* is the Huang–Rhys parameter, ℏω is the energy of effective phonon, and *k* is Boltzmann’s constant.

From the fitting, we have obtained the value of the Huang–Rhys factor for Cr^3+^ and Cr^4+^, *S* = 6.8 ± 0.2 and 3.6 ± 0.1, respectively, and effective phonon energy, *ℏω* = 310 ± 6 cm^–1^ for Cr^3+^, and 402 ± 7 cm^–1^ for Cr^4+^. Quantity *S*ℏω is the energy of electron lattice relaxation whose calculated value equals 2106 ± 40 and 1447 ± 25 cm^–1^ for Cr^3+^ and Cr^4+^, respectively. The magnitude of the electron–lattice coupling can be described by the Stokes shift, which is equal to *2S*ℏω. This parameter describes the energy difference between PLE and PL maximum bands (indicated by arrows in Figure 2a,b, cyan for Cr^3+^ and magenta for Cr^4+^). The *S*ℏω calculated from PLE and PL spectra for Cr^3+^ is 1715 ± 300 cm^–1^, which is smaller than those calculated from temperature-dependent *fwhm*. In the case of Cr^4+^, *S*ℏω is equal to 1415 ± 240 cm^–1^, which agrees with those obtained from temperature-dependent *fwhm*. The obtained parameters allow us to construct the one-dimensional configurational coordinate diagrams of the ground state (represented by black parabola) and excited states (represented by color parabolas) of Cr^3+^ and Cr^4+^ presented in Figure 4e,f. The *S*ℏω values were taken from the temperature-dependent *fwhm*. It should be noted that the results discussed above refer to the *x* = 0.10 sample. However, the relatively small changes observed in the PLE and PL spectra suggest that similar diagrams would be accepted for the other considered samples.

Figure 4d shows the temperature-dependent Cr^3+^ and Cr^4+^ emission intensity of *x* = 0 (empty dots) and 0.10 (filed dots) samples. In both samples, the emission intensity of Cr^3+^ increases to 275 K and then decreases at higher temperatures. In contrast, for Cr^4+^_,_ the emission begins to diminish significantly above 100 K. The slight increase in Cr^3+^ emission intensity can be interpreted as an increase in the absorption coefficient, which is related to the increasing phonon population.^58^ The experimental data of temperature dependence of Cr^3+^ emission intensity were fitted in the temperature range ≥275 K, while for Cr^4+^ in all temperature ranges. The following formula, inclusive of the single deexcitation process, was used to describe the temperature-dependent intensity behavior:
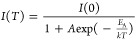
5where *I*_0_ is the PL intensity at 0 K, *E*_*A*_ is the activation energy, and *A* is the relative probability of the nonradiative deexcitation processes. The fitting curves are represented as solid lines in Figure 4d. The obtained parameters for *x* = 0 and 0.10 are as follows: *A* = 4070 ± 900; 3270 ± 1760, and *E*_A_ = 2540 ± 70 cm^–1^; 2760 ± 170 cm^–1^, respectively. The activation energy corresponds to nonradiative quenching of Cr^3+^ luminescence. The activation energy *E*_A_ and parameter *A* for Cr^4+^ ions (roughly the same for both samples) are 530 ± 45 cm^–1^ and 150 ± 50, respectively. This activation energy represents the nonradiative quenching of Cr^4+^ luminescence, which is approximately five times smaller than that of Cr^3+^. Considering the configuration diagrams, we can certainly say that the nonradiative processes are not solely attributed to the thermally induced nonradiative relaxation process directly from the excited state to the ground state: ^4^T_2_ → ^4^A_2_ transition in the case of Cr^3+^ and ^3^T_2_ → ^3^T_1_ transition for Cr^4+^. The intersections of the ground and excited states, represented by parabolas, occur at much higher energies for Cr^3+^ (∼18,000 cm^–1^) and Cr^4+^(∼9000 cm^–1^), which are much greater than that obtained from fitting the temperature dependence of intensity (2540–2760 cm^–1^).

Figure S6 shows the temperature dependence of luminescence kinetics in the μs range. Figure S6a,b shows the luminescence decay of Cr^3+^ emission upon excitation at 470 nm for *x* = 0 and 0.10 samples, respectively. Figure S6c shows the luminescence decay time of Cr^4+^ emission upon excitation at 980 nm. Due to the nonexponential decay, the average decay time was calculated using eq 3. Figure S6d,e shows the temperature dependence of the average decay time of Cr^3+^ and Cr^4+^ luminescence. The decay time values for Cr^3+^ at 10 K for *x* = 0 and 0.10 are 27 and 37 μs, respectively. For Cr^4+^, the decay time at 100 K is 1.34 ms. The decay time of Cr^3+^ is typical of Cr^3+^ in weak crystal fields.^49,59,60^ Similar decay time of Cr^4+^ was found in the references.^32^ For *x* = 0.10, the decay times of Cr^3+^ emissions remain stable up to 300 K and then decrease with increasing temperature. The temperature behavior of decay times aligns with the emission intensity behavior (Figure 4d). In the case of the *x* = 0 sample, where the energy transfer is more prominent, the decay times decrease slightly up to 300 K, followed by a more rapid decrease. The significant reduction in the decay time is related to nonradiative quenching of Cr^3+^. The shorter decay time observed at low temperatures for the *x* = 0 sample is attributed to an energy transfer process. The decrease in the decay time observed for Cr^4+^ emission is associated with nonradiative quenching. In both cases, the process of luminescence quenching remains unclear. However, we can certainly say that it is not solely attributed to the thermally induced nonradiative relaxation process directly from the excited state to the ground state (parabolas intercrossing). Instead, it could be attributed to either an ionization transition to the conduction band (specifically in the case of Cr^3+^) or defects or closely lying charge transfer (CT) states. In contrast, for Cr^4+^, these states are situated deeper within the bandgap, eliminating the possibility of an autoionization process.

### Pressure-Dependent Photoluminescence

**Figure 5:**
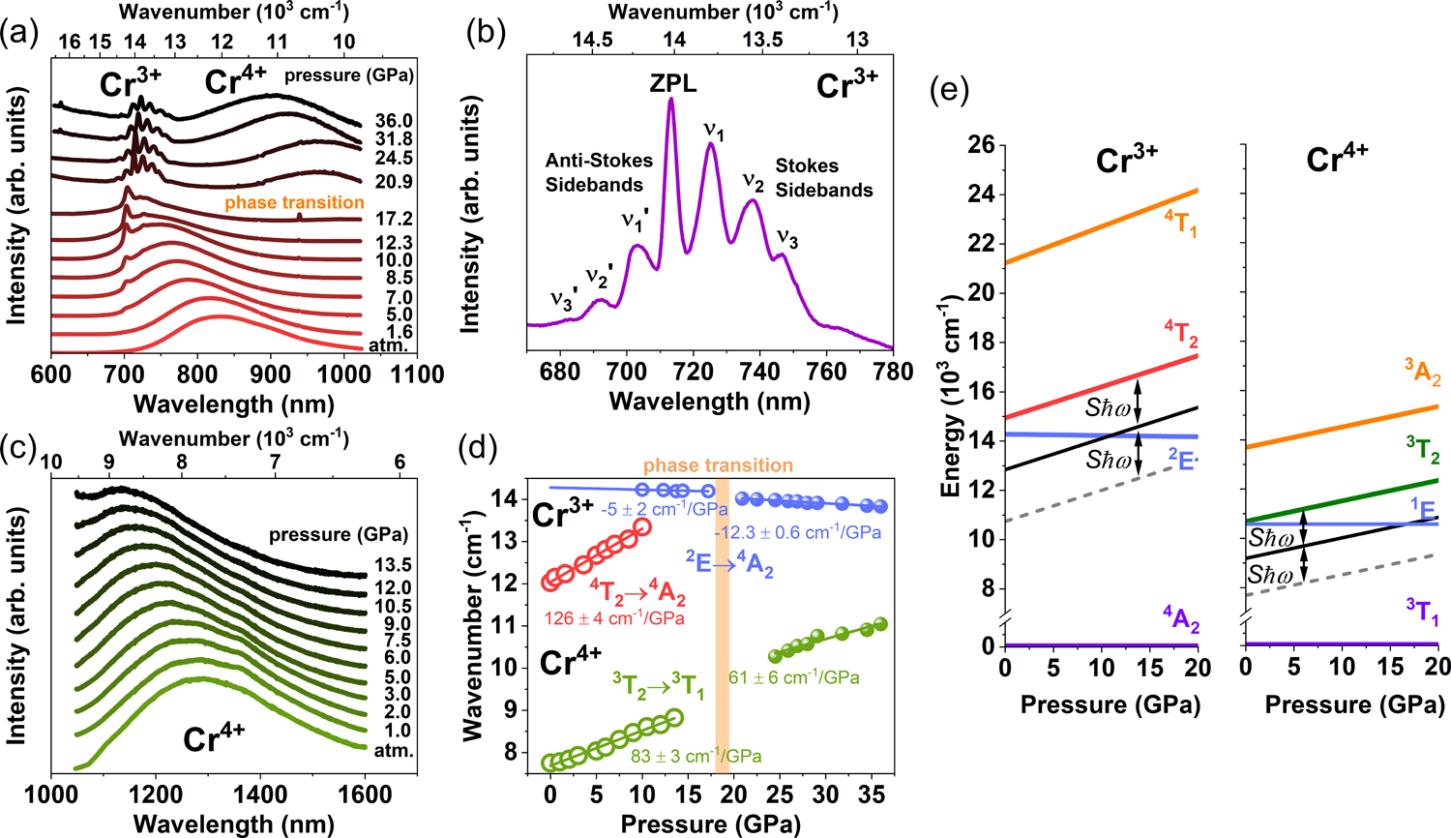
Pressure dependence of the luminescence of Cr^3+^ and Cr^4+^ in SGOC, *x* = 0.10. (a) Normalized RT emission spectra at different pressure for Cr^3+^ emission excited with 473 nm. (b) Cr^3+^ luminescence after a phase transition. (c) Normalized RT emission spectra at different pressures for Cr^4+^ emission excited with 980 nm. (d) Pressure-dependent emission shift in the energy scale. (e) Schematic pressure dependence of the selected energy levels of the Cr^3+^ and Cr^4+^ ions before phase transition. Black solid and dashed gray lines are related to the energy of the ^4^T_2_ level reduced by *S*ℏω and 2*S*ℏω, respectively.

To further understand the crystal field influence on Cr^3+^ and Cr^4+^ ion luminescence in SGOC, high-pressure experiments were conducted. The *x* = 0.10 sample was selected for further study due to its consistent demonstration of the highest luminescence intensity of Cr^3+^ ions among all of the samples containing a pure phase of Sc_2_O_3_. RT pressure-dependent PL spectra of Cr^3+^ and Cr^4+^ for *x* = 0.10 are presented in Figure 5a,c, respectively. The excitation wavelength for Cr^3+^ luminescence was 473 nm, while for Cr^4+^, it was 980 nm. At atmospheric pressure, only broadband emissions are simultaneously observed for Cr^3+^ and Cr^4+^ luminescence. The strong dependence of the ^4^T_2_ state of Cr^3+^ ions on crystal field strength leads to a substantial increase in the energy of the ^4^T_2_ → ^4^A_2_ transition with pressure and a slight decrease in the energy of 2E → 4A2 transition,^24^ as shown in the schematic energy structure diagram present in Figure 5e (left panel). For Cr^3+^ luminescence, the maximum broadband (^4^T_2_ → ^4^A_2_) emission spectra shift toward a shorter wavelength (higher energy). Above 12.3 GPa, the broadband emission disappears, and only narrow line emission is observed. This is explained by the crossing of two energy levels: ^4^T_2_ (black line) and ^2^E (blue line), as shown in Figure 5e, left panel (the energy levels represented by the black line is related to the energy of the ^4^T_2_ level reduced by *S*ℏω, while the dashed line by 2*S*ℏω). At room temperature, the thermal energy is too low to induce emission from the ^4^T_2_ state, and the lowest excited ^2^E state becomes the only emitting state. A similar effect is described in the references.^61−63^ At a pressure of 20.2 GPa, a change in emission spectra is observed. A phase transition likely causes such phenomena.^43−45^ It is worth noting that the new phase is metastable and the phase no longer persists when the pressure is released.

The Cr^3+^ emission in the new phase consists of seven well-resolved emission lines at 681.7, 691.9, 703.0, 713.3, 725.1, 737.5, and 746.6 nm, which are clearly shown in Figure 5b. The line at 713.3 is likely the zero phonon line (ZPL), while the lines at shorter wavelengths are anti-Stokes phonon sidebands marked as υ_1_’- υ_3_’, and the line at longer wavelengths (lower energies) are Stokes phonon sidebands marked as υ_1_- υ_3_. Surprisingly, we obtained well-resolved emission spectra at RT and high pressures after the phase transition. That emission spectrum is unusual for Cr^3+^ ions and can be found mostly for Mn^4+^-doped fluorides^11,64^ but not found for Cr^3+^ emission so far. The most similar Cr^3+^ spectrum can be found in the high symmetric structure of ZnGa_2_O_4_:Cr^3+^^65^ and K_2_NaGaF_6_:Cr^3+^,^66^ but it is still far from what is observed in this paper. This suggests that Cr^3+^ in the new phase is in a high-symmetry environment.

As mentioned in the Introduction section, there are three main phases found in rare-earth sesquioxide: hexagonal (*P*3*m*1); monoclinic, (*C*2*/m*); and cubic (*Ia*3̅).^39−41^ Another structure of sesquioxide found in the literature is a corundum-like structure (trigonal, *R*3̅*c*).^43^ The two other phases hexagonal (*P*6_3_*/mmc*) and cubic (Im3̅*m*) are formed at very high temperatures.^42^

The Sc_2(1–*x*)_Ga_2*x*_O_3_:Cr^3+/4+^ sample synthesized in this work adopts a cubic structure, with a space group *Ia*3̅. Among the listed structures, only a monoclinic arrangement with the space group *C2/m*, featuring either six or seven-coordinated cation sites, or a trigonal structure (*R*3̅*c*) with six-coordinated cation sites, is the viable candidate for the obtained phases. In the other structures, the requisite 6-fold coordination sites are where the Cr^3+^ ions can be located. Interestingly, the luminescence behavior of Cr^3+^ in similar materials (such as β-Ga_2–*x*_Sc_*x*_O_3:_Cr^3+^ with a monoclinic *C*2*/m* structure) and corundum-like structures is reported in the literature.^8,24,67^ However, these materials exhibit emissions different from those observed in this study. This discrepancy underscores that the exact origin of unusual Cr^3+^ emission following phase transition remains not fully understood.

The energies of the ^4^T_2_ and ^2^E transition to the ^4^A_2_ ground state of Cr^3+^ versus pressure before and after the phase transition are presented in Figure 5d. As pressure increases, the ZPL lines shift toward a longer wavelength (lower energy). The values of pressure shifts before phase transition were estimated for the ZPL (^2^E → ^4^A_2_) and broadband emission (^4^T_2_ → ^4^A_2_) and are −5 ± 2 and 126 ± 1 cm^–1^/GPa, respectively, which are typical for the pressure shifts of these transitions.^24,59,61−63,68^ The pressure shift of the Stokes and anti-Stokes phonons of the Cr^3+^ emission of the new high-pressure phase is shown in Figure S7. It is seen that the pressure shift is roughly the same for all emission lines. The ZPL shift rate after the phase transition is estimated to be −12.3 ± 0.1 cm^–1^/GPa.

The schematic energy structure diagram for Cr^4+^ ions is presented in Figure 5e (right panel). The pronounced sensitivity of the ^3^T_2_ state of Cr^4+^ ions to the crystal field strength results in a significant rise in the energy of the ^3^T_2_ → ^4^T_1_ transition under pressure, along with a minor reduction in the energy of the ^1^E → ^4^T_1_ transition, similar to Cr^3+^ ions. For Cr^4+^ luminescence, a linear shift of the broadband emission toward higher energies is observed with increasing pressure. The calculated pressure shift is presented in Figure 5d. It is worth noting that only a few studies in the literature have conducted high-pressure investigations on NIR Cr^4+^ emission.^31,33,35^ The linear shift of Cr^4+^ is estimated to be 83 ± 3 and 61 ± 6 cm^–1^/GPa before and after the phase transition, respectively. Although we conducted a high-pressure experiment up to 36 GPa, we did not reach the pressure value and observed the crossover of the ^3^T_2_ and ^1^E states for Cr^4+^ in RT. Although the schematic energy diagram indicates a crossing between ^3^T_1_ and ^1^E states around 20 GPa, as shown in Figure 5e, this phenomenon is not observed in RT pressure dependence of emission spectra. Moreover, as pressure increases further, a phase transition takes place. It is important to note that the schematic diagram shown in Figure 5e will no longer accurately represent the energy diagram of the sample in the new phase.

Figure S8a in the SI shows the pressure-dependent decay profiles of Cr^3+^ luminescence up to 25 GPa and upon excitation at 473 nm. The pressure-dependent decay profiles were taken from the whole emission range (taken from the maximum of the luminescence band, they show a similar trend). The decay profiles are multiexponential, and the average decay times τ_av_ were calculated using eq 3. The calculated decay time values are presented in Figure S8b. Pressure-induced increase of crystal field strength causes an increase in the energetic separation of ^4^T_2_ and ^2^E excited states as shown in Figure 5e, lowering the thermal occupation of the higher ^4^T_2_. Since the thermal occupation of the ^4^T_2_ state with μs lifetime affects the adequate decay time of the ^2^E → ^4^A_2_ emission, the increase of pressure leads to rapid elongation of the decay time of luminescence.

## Conclusions

A series of new Sc_2(1–*x*)_Ga_2*x*_O_3_:Cr^3+/4+^ materials with *x* = 0–0.20 were synthesized, exhibiting broadband near-infrared (NIR-I and NIR-II) emission. X-ray powder diffraction reveals the absence of impurity peaks for *x* = 0–0.05. The diffraction peak position shift toward higher angles was observed for *x* = 0–0.10 indicating the incorporation of Ga^3+^ ions into the Sc_2_O_3_ structure. However, no further shifts were observed for *x* = 0.10–0.20, suggesting that Ga^3+^ could no longer be incorporated into the Sc_2_O_3_ structure. In a previous study, the pure phase of Ga_2(1–*y*)_Sc_2*y*_O_3_:Cr^3+/4+^ was achieved when the Sc^3+^/Ga^3+^ ratio was lower than 44%, originating from the Ga_2_O_3_ structure. Conversely, the pure phase of Sc_2(1–*x*)_Ga_2*x*_O_3_ was obtained from the Sc_2_O_3_ structure when the Sc^3+^/Ga^3+^ ratio exceeded 94%. The crystal structure of Sc_2(1–*x*)_Ga_2*x*_O_3_ consists of two 6-fold coordinated Sc^3+^ sites (Sc1 and Sc2), both exhibiting distortion, but the distortion of Sc2 sites is much more significant. Excitation at 450 nm effectively stimulates the studied phosphors, suggesting their potential application in NIR-LEDs. Under 490 nm excitation, Sc_2(1–*x*)_Ga_2*x*_O_3_:Cr^3+/4+^ demonstrates an ultra-broadband NIR emission band ranging from 650 to 1100 nm and 1100 to 1600 nm, originating from Cr^3+^ and Cr^4+^ ions, respectively, matching the NIR-I and NIR-II regions. The RT *fwhm* value for Cr^3+^ is 2402 cm^–1^, while for Cr^4+^ ions, it is 2075 cm^–1^. The strong distortion of the 6-fold coordinated Sc2 site suggests that Cr^4+^ ions may occupy this site. The intensity of Cr^3+^ emission increases significantly (around 77 times) from *x* = 0 to *x* = 0.20. This finding suggests the positive impact of Ga^3+^ on the enhancement of the luminescent properties of the material. Energy transfer between Cr^3+^ and Cr^4+^ ions occurs in the studied materials. Taking into account the configuration diagrams presented in this paper, we can certainly say that the nonradiative processes do not result from the thermally induced nonradiative relaxation process directly from the excited state ^4^T_2_ to the ground state ^4^A_2_. Instead, these processes could potentially be ascribed to either an ionization transition to the conduction band (specifically in the case of Cr^3+^) or the presence of defects or closely lying charge transfer (CT) states. Furthermore, a phase transition is observed at a pressure of 20.2 GPa. The emission spectra obtained after phase transition exhibit high resolution at high pressures, indicating that Cr^3+^ in the new phase exists in a high-symmetry environment. That emission spectrum is unusual for Cr^3+^ ions, and the origin of Cr^3+^ emission following the phase transition remains not fully understood. Additionally, we present the pressure-induced shift of the NIR Cr^4+^ luminescence in Sc_2(1–*x*)_Ga_2*x*_O_3_:Cr^3+/4+^. The linear shifts are estimated to be 83 ± 3 cm^–1^/GPa and 61 ± 6 cm^–1^/GPa before and after phase transition, respectively.

## Experimental Section

Gallium oxide (Ga_2_O_3_, 99.99%), scandium oxide (Sc_2_O_3_, 99.99%), and chromium oxide (Cr_2_O_3_, 99.99%) were purchased from Gredmann. To prepare the Sc_2(1–*x*)_Ga_2*x*_O_3_:Cr^3+^ samples, all the precursors were stoichiometrically weighed and mixed with an agate mortar for 30 min. The mixing precursors were then poured into alumina crucibles and placed in a muffle furnace. Then, all the samples were heated to 1200 °C for 5 h, with a heating and cooling rate of 5 °C/min. After the samples were cooled to room temperature, they were ground, and the final products were obtained.

## Supporting Information Available

Characterization; crystal structure; X-ray diffraction; Rietveld refinement; photoluminescence; and decay time (PDF)
