# Supplementary material for: Advancing Near-Infrared Light Sources: Enhancing Chromium Emission through Cation Substitution in Ultra-Broadband Near-Infrared Phosphors
Source: Chem Mater. 2023 Nov 17;35(23):10228–37. doi: 10.1021/acs.chemmater.3c02466 (PMC10720341; doi:10.1021/acs.chemmater.3c02466)
Supplement: Supplementary file 1 — cm3c02466_si_001.pdf [file cm3c02466_si_001.pdf]

Supporting Information

Advancing Near-Infrared Light Sources: Enhancing Chromium Emission through Cation Substitution in Ultra-Broadband Near-Infrared Phosphors

Natalia Majewska,[Ψ] Yi-Ting Tsai,[†] Xiang-Yun Zeng,[†] Mu-Huai Fang[†,*] and Sebastian Mahlik,[Ψ, §,*]

[Ψ]Institute of Experimental Physics, Faculty of Mathematics, Physics and Informatics, University of Gdansk, Wita Stwosza 57, 80-308 Gdansk, Poland

[†]Research Center for Applied Sciences, Academia Sinica, Taipei 11529, Taiwan

[§]International Centre for Theory of Quantum Technologies (ICTQT), University of Gdansk, 80-308 Gdansk, Poland

Characterization

Synchrotron Powder X-ray diffraction patterns are characterized on the National Synchrotron Radiation Research Center (NSRRC, Taiwan) with BL19A1 beamline at room temperature (RT) under the energy and wavelength of 20 keV and 0.61992 Å, respectively. The synchrotron diffraction patterns were used for Rietveld refinement by the Total Pattern Analysis Solutions software (TOPAS 6.0). The X-ray absorption of Cr was measured by Cr *K*-edge X-ray absorption near-edge structure (XANES) and extended X-ray absorption fine structure (EXAFS) at the BL44A1 beamline in NSRRC.

RT photoluminescence excitation (PLE) spectra of Cr^{3+} (near-infrared emission, NIR-I) were recorded with a FluoroMax-4P spectrofluorometer (Horiba). The spectrofluorometer is equipped with a 150 W xenon lamp for excitation and an R928 Hamamatsu photomultiplier as a detector. A custom-made setup was utilized to measure the PLE of longer-wavelength near-infrared (NIR-II) emission from Cr^{4+}. The setup consists of an excitation source, which includes an EQ99X laser-driven Xe light source (Energetiq) coupled to a self-made grating monochromator operating in the 250-1000 nm range. The luminescence detection was carried out using an Andor SR-500i-D1 spectrometer equipped with a CCD camera (DU490A-1.7). The spectrometer operated in the 600-1700 nm wavelength range.

The temperature and pressure-dependent NIR-I emission (Cr^{3+}) spectra were measured using an Andor SR-750-D1 spectrometer equipped with a CCD camera (DU420A-OE). The 473 nm laser and 470 nm diode were used as the excitation sources.

The temperature and pressure-dependent NIR-II emission (Cr^{4+}) spectra were captured using an Andor SR-500i-D1 spectrometer. The spectrometer is equipped with a CCD camera (DU490A-1.7) operating in the 600-1700 nm wavelength. The 980 nm laser was utilized as the excitation source.

The decay profiles of NIR-I and NIR-II emissions were measured using time-resolved spectroscopy apparatus. The setup comprises a PG 401/SH optical parametric generator pumped by a PL2251A pulsed YAG:Nd laser (EKSPLA). A 2501S grating spectrometer (Bruker Optics) was combined with a C4334-01 streak camera (Hamamatsu) to detect NIR-I emission. The data were recorded as streak images using a CCD array with dimensions of 640 by 480 pixels. The recorded streak images were processed using a software-based photon counting algorithm. This algorithm transformed the raw data into a two-dimensional (2D) matrix representing photon counts with respect to wavelength and time. (streak image).[1] In the case of NIR-II emission, the detection setup involved the use of an Avalanche Photodetector APD110C/M that operates within the spectral range of 900-1700 nm. This detector was combined with a National Instrument Data Acquisition module.

To study temperature-dependent NIR-I and NIR-II emission and NIR-II decay profiles, precise temperature control was achieved using the THMS600 temperature controller Linkam stage in conjunction with the LNP95 liquid nitrogen cooling pump system. This setup enabled measurements to be performed over a wide temperature range of 77-600 K. For temperature-dependent NIR-I decay profiles, and the samples were cooled using an APD Cryogenics closed-cycle DE-202 optical cryostat. This cryostat provided a controlled cooling system, allowing the temperature to be varied within the range of 10 to 450 K.

The high-pressure luminescence measurements were performed using a screw-driven Merrill-Bassett-type diamond anvil cell. The diamond anvil cell has a 0.5 mm diamond culet diameter.[2] The gasket for the pressure chamber was pre-indented to around 0.07 mm. In the center of the pre-indentation, a hole with a diameter of 0.22 mm was drilled. The $KMgF_3$:0.5%Eu^{2+} was used as a pressure sensor,[3] because it exhibits luminescence in the UV range and does not emit in the Cr^{3+} ion luminescence range. Polydimethylsiloxane oil providing quasi-hydrostatic pressure up to 40 GPa has been used as a pressure-transmitting medium. We evaluated measurement consistency to guarantee the reliability of our measurements and also investigated the possible impact of this quasi-hydrostatic environment on phase transitions. Our findings demonstrate that our measurements are consistent, consistently delivering comparable pressure values for phase transitions in each iteration of our high-pressure experiments.

Results and Discussion

Structural Analysis

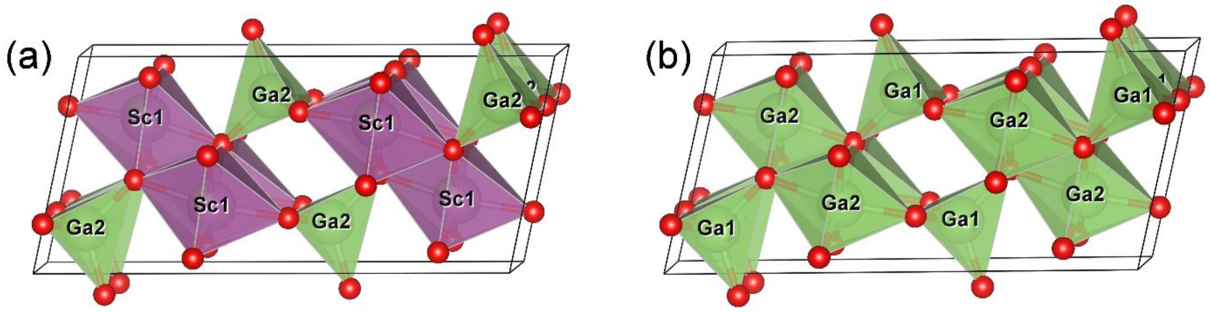

Figure S1. Crystal structure of (a) $Ga_{1.17}Sc_{0.83}O_3$ and (b) Ga_2O_3.

Table S1. Information of Sc1 and Sc2 in $[ScO_6]$ polyhedrons extracted from the crystallographic information framework of Sc_2O_3.

	**Sc1**	**Sc2**
Polyhedron	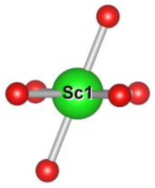	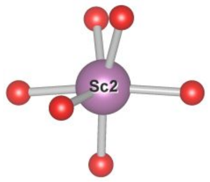
Chemical bonds	(Sc1–O1) = 2.10314(0) Å<br>(Sc1–O1) = 2.10314(0) Å<br>(Sc1–O1) = 2.10314(0) Å<br>(Sc1–O1) = 2.10314(0) Å<br>(Sc1–O1) = 2.10314(0) Å<br>(Sc1–O1) = 2.10314(0) Å	(Sc2–O1) = 2.12(7) Å<br>(Sc2–O1) = 2.12(7) Å<br>(Sc2–O1) = 2.08(4) Å<br>(Sc2–O1) = 2.10(6) Å<br>(Sc2–O1) = 2.10(6) Å<br>(Sc2–O1) = 2.08(4) Å
Average bond length	2.1031 Å	2.0978 Å
Polyhedral volume	11.2948 $Å^3$	10.8330 $Å^3$
Distortion index	0	0.00604
Bond angle variance	180.5362 $(degree)^2$	246.4595 $(degree)^2$

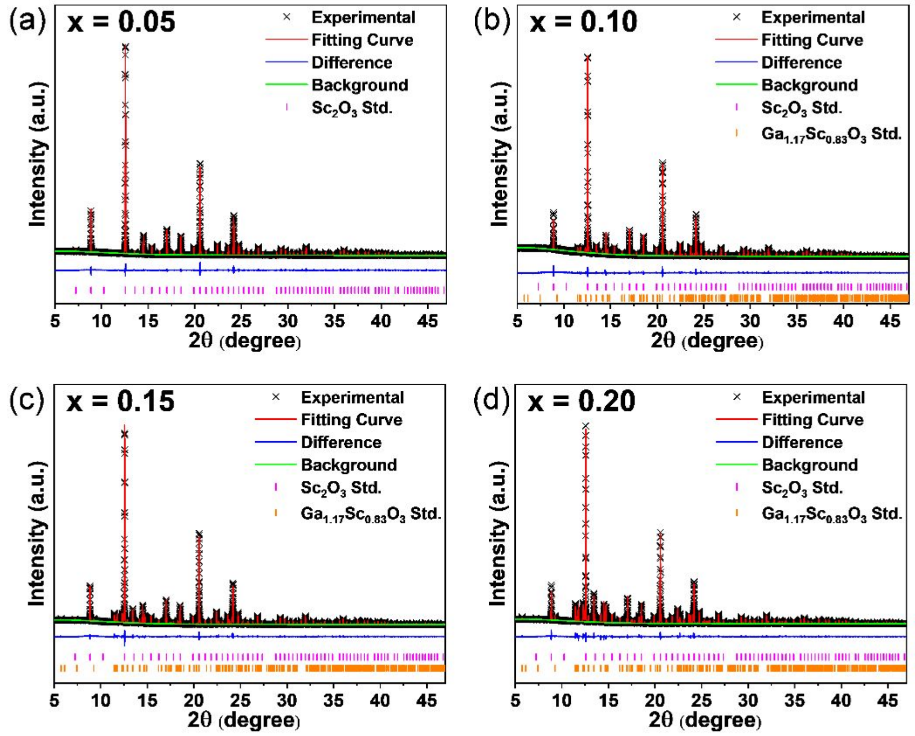

Figure S2. Rietveld refinement of SGOC with (a) x = 0.05, (b) x = 0.10, (c) x = 0.15, and (d) x = 0.20.

Table S2. Refined parameters of SGOC with x = 0–0.2.

x	0	0.05	0.10	0.15	0.20
a (Å)	9.84121(2)	9.82168(2)	9.81542(3)	9.81575(2)	9.81643(3)
V (Å^3)	953.116(6)	947.451(7)	945.641(8)	945.738(6)	945.933(8)
GOF	1.21	1.94	1.65	1.67	1.8
R_{wp} (%)	3.88	5.44	4.79	3.88	3.74
R_p (%)	3.09	4.02	3.68	2.9	2.68

Table S3. Atomic positions, occupancies, and atomic displacement parameters of SGOC with x = 0–0.2.

x = 0						
Site	Np	*x*	*y*	*z*	*Occ*	*Beq*
Sc1	8	0.25	0.25	0.25	1	0.000(6)
Sc2	24	0.96439(2)	0	0.25	1	0.000(4)
O1	48	0.39102(7)	0.15445(6)	0.38190(7)	1	0.040(11)

x = 0.05						
Site	Np	*x*	*y*	*z*	*Occ*	*Beq*
Sc1	8	0.25	0.25	0.25	0.956(5)	0.023(13)
Ga1	8	0.25	0.25	0.25	0.044(5)	0.023(13)
Sc2	24	0.96468(3)	0	0.25	0.962(4)	0.039(8)
Ga2	24	0.96468(3)	0	0.25	0.038(4)	0.039(8)
O1	48	0.39123(11)	0.15480(10)	0.38177(11)	1	0.12(3)

x = 0.15						
Site	Np	*x*	*y*	*z*	*Occ*	*Beq*
Sc1	8	0.25	0.25	0.25	0.956(6)	0.034(16)
Ga1	8	0.25	0.25	0.25	0.044(6)	0.034(16)
Sc2	24	0.96487(4)	0	0.25	0.959(5)	0.073(10)
Ga2	24	0.96487(4)	0	0.25	0.041(5)	0.073(10)
O1	48	0.39112(12)	0.15478(12)	0.38190(13)	1	0.22(3)

x = 0.1						
Site	Np	*x*	*y*	*z*	*Occ*	*Beq*
Sc1	8	0.25	0.25	0.25	0.942(7)	0.240(19)
Ga1	8	0.25	0.25	0.25	0.058(7)	0.240(19)
Sc2	24	0.96470(4)	0	0.25	0.944(6)	0.258(11)
Ga2	24	0.96470(4)	0	0.25	0.056(6)	0.258(11)
O1	48	0.39113(15)	0.15414(14)	0.38261(16)	1	0.36(4)

x = 0.20						
Site	Np	*x*	*y*	*z*	*Occ*	*Beq*
Sc1	8	0.25	0.25	0.25	0.950(7)	0.00(2)
Ga1	8	0.25	0.25	0.25	0.050(7)	0.00(2)
Sc2	24	0.96489(4)	0	0.25	0.967(5)	0.001(12)
Ga2	24	0.96489(4)	0	0.25	0.033(5)	0.001(12)
O1	48	0.39108(14)	0.15462(14)	0.38210(16)	1	0.16(4)

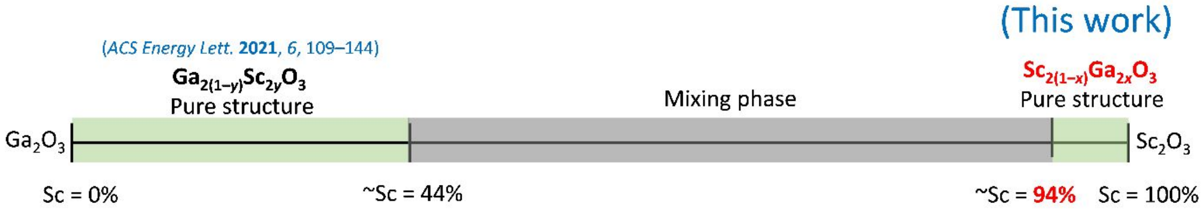

Figure S3. Scheme of phase evolution with different Sc^{3+} concentrations in $Sc_{2(1-x)}Ga_{2x}O_3$.

Photoluminescence

Crystal field and Racah parameters calculation

Due to the large bandwidth in optical spectra associated with lattice relaxation for the $^4A_2 \rightarrow {}^4T_1$ and $^4A_2 \rightarrow {}^4T_2$ optical transitions, the selection of transition energy for calculating crystal field parameters is not trivial and comes with significant uncertainty, as shown in Figure S4 b. Furthermore, the position of the ^2E state cannot be readily estimated from the excitation and emission spectra at ambient conditions. The energy of the ^2E state was calculated to be 14288 cm^{-1} by extrapolating the linear fitting from the observed shift of the $^2E \rightarrow {}^4A_2$ transition at high pressure for $x = 0.1$ (Figure 5c in the main manuscript).

Knowing the location of the $^4T_2$, $^4T_1$, and ^2E states of Cr^{3+} ions (for x =0.1) enables us to calculate crystal field parameter Dq, which describes the mutual influence between the 3d electrons and ligand ions, as well as the Racah parameters B and C, which represent the

interaction between 3*d* electrons in Cr^{3+}. The $^4A_2 \rightarrow ^4T_2$ transition energy of the maximum excitation band is equal to 10*Dq*. Racah parameters *B* and *C* can be calculated from the following equations: [4]

$$B = Dq\frac{\left[\frac{\Delta E}{Dq}\right]^2 - 10 \cdot \frac{\Delta E}{Dq}}{15\left(\frac{\Delta E}{Dq} - 8\right)}, \tag{S1}$$

$$C = \frac{B}{3.05}\left\{\frac{E(^2\mathrm{E})}{B} - 7.9 + 1.8\frac{B}{Dq}\right\}, \tag{S2}$$

where: ΔE is the difference between the energy of the $^4A_2 \rightarrow ^4T_1$ and $^4A_2 \rightarrow ^4T_2$ transitions. Since we have information about the energy of the ^2E state only for the $x = 0.1$ sample, the aforementioned parameters were calculated only for the $x = 0.1$ sample. However, the relatively small changes observed in the PLE and PL spectra suggest that a similar value of *Dq*, *B,* and *C* parameters would be applicable to the other samples under consideration. The value of the $^2E \rightarrow ^4A_2$ transition was derived from the linear fitting of the ^2E state position versus pressure (refer to Figure 5 c in the main manuscript). The energies of the $^4A_2 \rightarrow ^4T_1$ and $^4A_2 \rightarrow ^4T_2$ transitions are obtained from the maximum of corresponding excitation bands. The *Dq*, *B,* and *C* calculated values are 1520, 596, and 3278 cm^{-1}, which agree with the values found in the literature for $Sc_2O_3{:}Cr^{3+}$.[5]

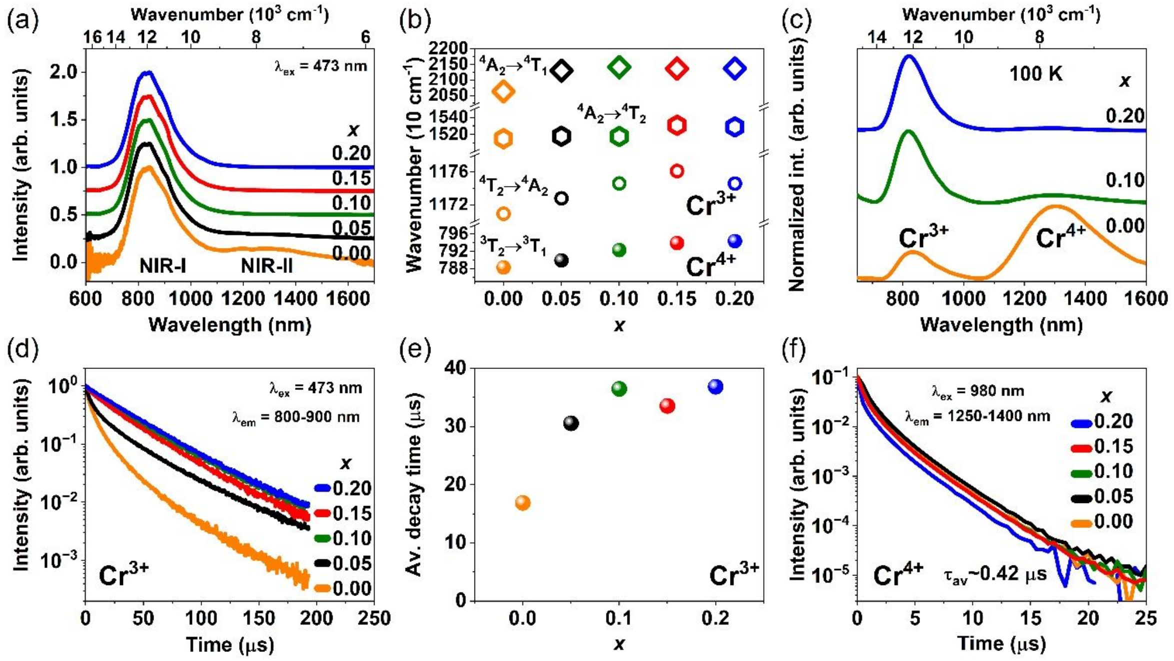

Figure S4. (a) Normalized room temperature emission spectra upon excitation at 473 nm. (b) Position of the maximum of the excitation band: $^4A_2 \rightarrow ^4T_1$ and $^4A_2 \rightarrow ^4T_2$; and emission bands: $^4T_2 \rightarrow ^4A_2$ of

Cr^{3+}, and the $^3T_2 \rightarrow {}^3T_1$ of Cr^{4+} vs x. (c) PL spectra of $x = 0$, 0.10, and 0.20 at 100 K. (d) The RT x dependence of (e) decay profiles and (f) the calculated average decay times of Cr^{3+} emission (f) Decay profiles of Cr^{4+} emission for $x = 0$ - 0.20 for SGOC. The errors fall within the range of the dot size.

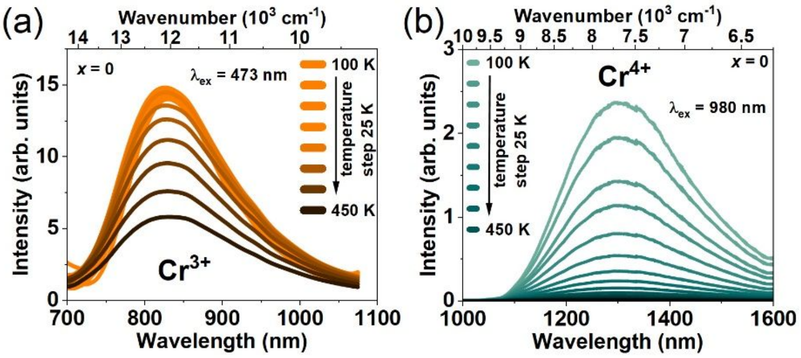

Figure S5. The temperature dependence of emission spectra (a) upon excitation at 473 nm for Cr^{3+} and (b) at 980 nm for Cr^{4+} of the $x = 0$ sample.

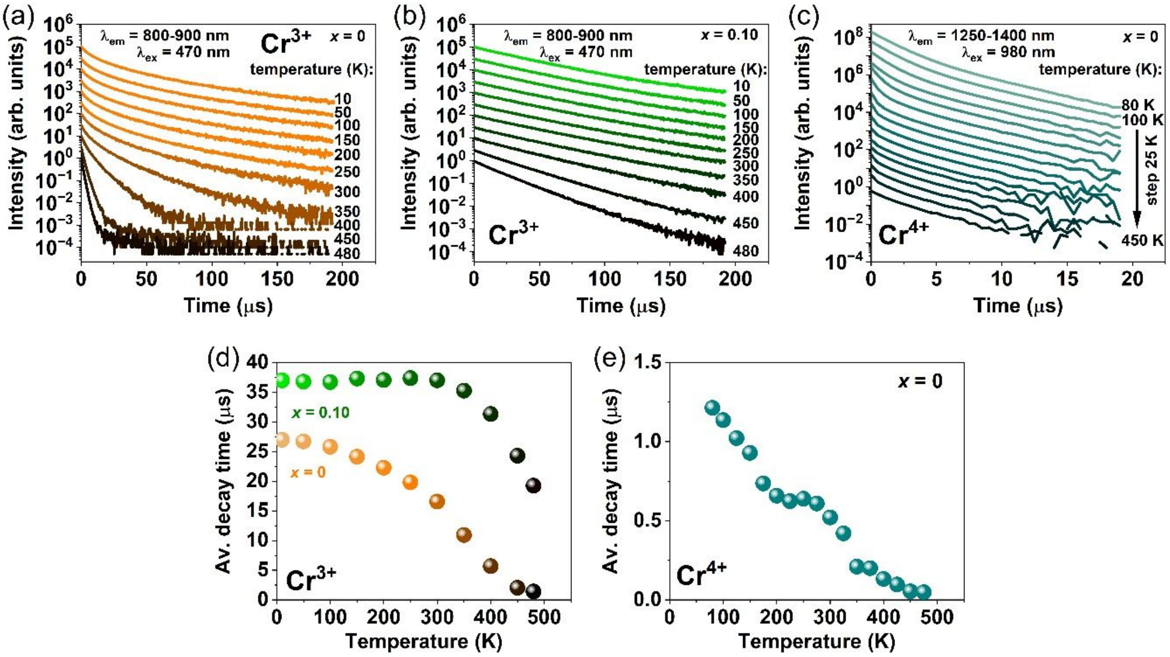

Figure S6. The temperature dependence decay profiles of Cr^{3+} for (a) $x = 0$ and (b) $x = 0.10$ upon excitation at 470 nm and (c) Cr^{4+} emission upon excitation at 980 nm. The average decay times of (d) Cr^{3+} and (e) Cr^{4+}. The errors fall within the range of the dot size.

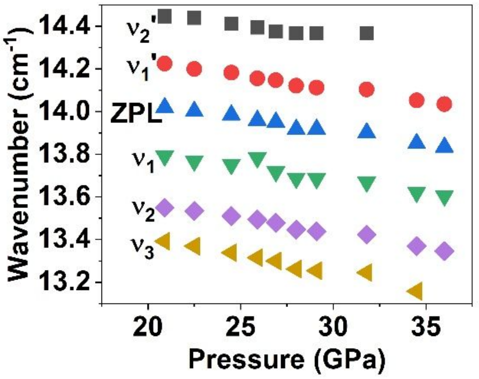

Figure S7. The pressure shift of zero phonon line (ZPL) and phonon Stokes (ν_1, ν_2 ν_3) and Anit-Stokes (ν_1', ν_2') sidebands. The errors fall within the range of the dot size.

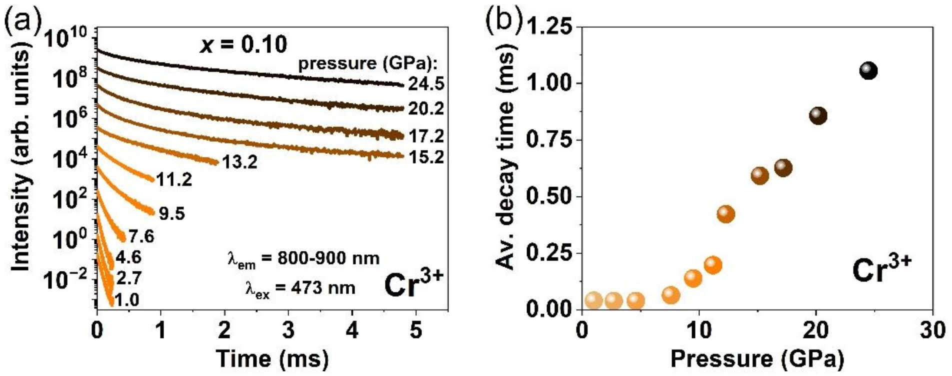

Figure S8. (a) Pressure dependence of decay profile for Cr^{3+} luminescence and (b) calculated average decay times of Cr^{3+} emission of x = 0.10 sample. The errors fall within the range of the dot size.

References:

(1) Merrill, L.; Bassett, W. A. Miniature Diamond Anvil Pressure Cell for Single Crystal X-ray Diffraction Studies. *Rev. Sci. Instrum.* **1974**, *45* (2), 290–294.

(2) Mahlik, S. High-Pressure Study of Phosphors Emission. In *Phosphor Handbook*; CRC Press, 2022.

(3) Barzowska, J.; Lesniewski, T.; Mahlik, S.; Seo, H. J.; Grinberg, M. $KMgF_3:Eu^{2+}$ as a New Fluorescence-Based Pressure Sensor for Diamond Anvil Cell Experiments. *Opt. Mater.* **2018**, *84*, 99–102.

(4) Henderson, B.; Imbusch, G. F. Optical Spectroscopy of Inorganic Solids; Monographs on the Physics and Chemistry of Materials; Oxford University Press: Oxford, New York, 2006.

(5) Brik, M. G.; Avram, N. M. Crystal Field Analysis and Electron-Phonon Coupling in $Sc_2O_3:Cr^{3+}$. *Z. Für Naturforschung A* **2004**, *59* (11), 799–803.
